# Effects of Sodium Alginate on the Flotation Separation of Molybdenite From Chalcopyrite Using Kerosene as Collector

**DOI:** 10.3389/fchem.2020.00242

**Published:** 2020-04-28

**Authors:** Guangsheng Zeng, Leming Ou, Wencai Zhang, Yuteng Zhu

**Affiliations:** ^1^College of Chemistry and Chemical Engineering, Central South University, Changsha, China; ^2^School of Minerals Processing and Bioengineering, Central South University, Changsha, China; ^3^Department of Mining and Minerals Engineering, Virginia Polytechnic and State University, Blacksburg, VA, United States

**Keywords:** molybdenite, chalcopyrite, sodium alginate, Cu^2+^-activation, flotation

## Abstract

In this paper, the effect of sodium alginate (SA) on the flotation separation of molybdenite (MoS_2_) from chalcopyrite using kerosene as collector was systematically investigated. The results of single-mineral micro-flotation tests indicated that SA exhibited strong depression on chalcopyrite flotation while it imposed no impact on the floatability of molybdenite. However, in the chalcopyrite–molybdenite mixed-mineral flotation system, the presence of chalcopyrite significantly increased the depressing effect of SA on molybdenite flotation, leading to a considerable reduction in the flotation selectivity. The negative impact of chalcopyrite on the performance of SA in molybdenite flotation was eliminated by adding a certain dosage of kerosene prior to SA. A concentrate containing 53.43% of molybdenum (Mo) was obtained at 76.90% of recovery using 19 mg/L kerosene and 40 mg/L SA at pH 5.4. Zeta potential measurements indicated that the adsorption of SA on chalcopyrite surfaces was stronger than that on molybdenite surfaces, which agreed with the single-mineral flotation test results. The adsorption of SA on chalcopyrite was further confirmed to be chemisorption by Fourier-transform infrared spectroscopy (FTIR) spectra analyses. When Cu^2+^ appeared in solution, the flotation of molybdenite was strongly depressed by SA. Mechanism analyses indicated that more active sites were generated on molybdenite surfaces after the addition of Cu^2+^, thus promoting the adsorption of SA.

## Introduction

Molybdenite (MoS_2_) is the primary source of molybdenum (Mo), and approximately half of the Mo production is obtained from copper (Cu)–Mo sulfides, mainly porphyry Cu deposits (Ansari and Pawlik, 2007; Song et al., 2012; Hirajima et al., 2014). At present, the flotation separation of Cu minerals and Mo minerals in this type of ore is normally achieved by selectively depressing the Cu sulfide minerals using various inorganic inhibitors. Sulfide is one of the most widely used Cu sulfide inhibitors in practical flotation processes (Pearse, 2005; Yin et al., 2010; Mehrabani et al., 2011; Zhao et al., 2018). However, the characteristics of high dosage and toxicity of inorganic inhibitors adversely affect the surrounding environment. Therefore, the development and application of more environment-friendly and cost-efficient inhibitors are crucial (Yin et al., 2017; Suyantara et al., 2018; Yuan et al., 2019a,b).

**Figure 1:**
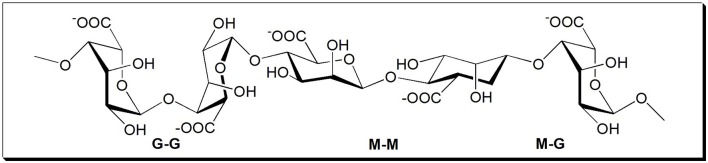
Molecular structure of sodium alginate (SA).

Sodium alginate (SA) is a natural non-toxic anionic polysaccharide extracted from various species of brown algae comprised of 1 → 4 linked β-D-mannuronic acid (M) and its C-5 epimer α-L-guluronic acid (G) (Wang and Peng, 2014). It has been extensively applied in the fields of medical, health, biology, food, etc. (Lin et al., 2005; Pawar and Edgar, 2012; Hu et al., 2017; Su et al., 2017; Li et al., 2019). The molecular structure of SA is shown in Figure 1. Its molecular structure contains a large number of hydroxyl (-OH) and carboxyl (-COO-) groups, and the gelling reaction of SA with divalent cations can occur easily in solution (Zhu et al., 2003; Liu et al., 2008; Yu et al., 2017). Based on the properties of SA described above, SA was considered to be a potential alternative to conventional inorganic inhibitors. The feasibility of using SA as a flotation inhibitor has been tested for various minerals. For instance, SA was used as an inhibitor of molybdenite using amyl xanthate as collector in chalcopyrite flotation (Chen et al., 2018) and as an inhibitor of galena using ammonium dibutyl dithiophosphate as collector in chalcopyrite (Chen et al., 2019) and as an inhibitor to separate the scheelite from calcite and fluorite (Chen et al., 2017). However, the effects of SA on the flotation performances of chalcopyrite and molybdenite using kerosene as collector have not been reported yet. Therefore, in this paper, SA was utilized as a depressant to separate the molybdenite from chalcopyrite using kerosene as the collector. The adsorption behaviors of SA on the mineral surfaces were studied by adsorption measurements, zeta potential measurements, and Fourier-transform infrared spectroscopy (FTIR) spectra analyses.

## Materials and Methods

### Mineral Samples and Reagents

**Figure 2:**
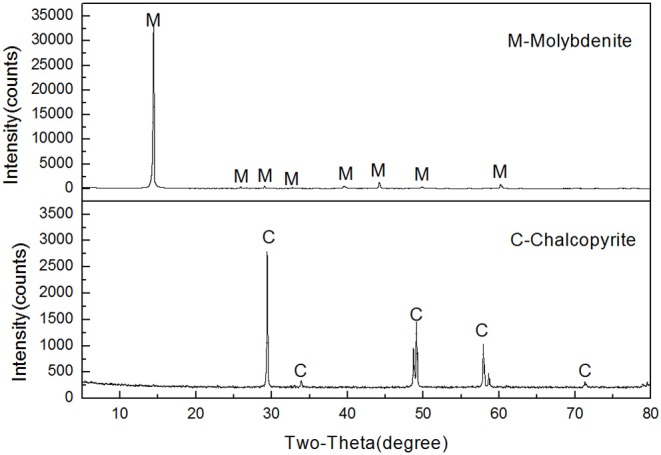
X-ray diffraction (XRD) spectra of the chalcopyrite and molybdenite samples.

Molybdenite and chalcopyrite samples used for this study were obtained from Tibet and Yunnan, China, respectively. High-grade mineral crystals were manually picked out and pulverized to a top size of 1 mm using a laboratory roll crusher and a ceramic ball mill. The product was dry-sieved with standard screens to acquire the −74 + 38-μm and −38-μm fractions. The former fraction was used for micro-flotation tests and adsorption measurements. A portion of the latter fraction was further ground to minus 2 μm for zeta potential measurements and FTIR analyses. Based on the X-ray diffraction (XRD) results shown in Figure 2, the purity of molybdenite and chalcopyrite was 95.68% and 98.23%, respectively.

SA was supplied by Enoson Biotechnology Co., Ltd., Wuhan, China. A stock solution of SA (0.04% by weight) was prepared by dispersing a required amount of SA in deionized water with high-speed stirring. Kerosene and CuCl_2_ were both purchased from Tianjin Kermil Chemical Reagents Development Centre, Tianjin, China. Hydrochloric acid (HCl) and sodium hydroxide (NaOH) were used as pH regulators and methyl isobutyl carbinol (MIBC) was used as frother. All reagents were analytical pure except for SA which was food grade. Deionized water was used throughout this study.

### Micro-Flotation Tests

All the flotation tests were conducted in an XFG-1600-type laboratory flotation machine. For single-mineral flotation tests, 2.0 g pure mineral sample was first placed in a beaker of 50-ml capacity together with 30 ml deionized water. Ultrasonic cleaning of 5 min was applied to the suspension to remove the oxidized substances on the mineral surface, after which the supernatant was decanted and the mineral particles were washed into a plexiglass flotation cell of 40-ml capacity with proper amount of deionized water. The pulp was stirred for 1 min at 1,902 rpm to ensure thorough mixing, after which the pH regulators, depressant (if needed), collector, and frother were sequentially added into the suspension. The pulp was conditioned for 3 min after each addition except for the frother (1 min). Flotation was conducted for a total period of 3 min for each test. After flotation, the concentrates (froth product) and tailings (in-tank product) were collected, filtered, dried and weighed. The recovery was calculated based on the weight distribution of mineral particles between the two products. Each single-mineral flotation test was repeated at least three times. The average values were reported in this study. Standard deviations were also calculated and presented as error bars.

For the mixed binary minerals flotation tests, the mass ratio of chalcopyrite and molybdenite was 2:1 (2.0 g chalcopyrite + 1.0 g molybdenite). The flotation procedures are similar to those used for the single-mineral flotation tests except that two different reagent addition sequences were tested, namely, kerosene prior to SA and SA prior to kerosene. The concentrates and tailings were assayed for Cu and Mo. The recovery of molybdenite and chalcopyrite was calculated based on the grades of Cu and Mo in the concentrates and tailings.

### Adsorption Measurements

The adsorption of SA on both the original and Cu^2+^-treated molybdenite was measured as a function of pH using a TOC-L machine supplied by Shimadzu, Kyoto, Japan. The detailed experimental procedures are as follows. First, 2.0 g of mineral samples were added to 40-ml aqueous solutions in the presence or absence of 15 mg/L Cu^2+^ followed by 30 min of conditioning. Then, the obtained suspensions were adjusted to the desired pH by adding hydrochloric acid and/or sodium hydroxide solutions, after which 40 mg/L SA was added into the suspensions followed by another 30 min of conditioning. The resultant suspensions were filtered, and subsequently the filtrate was centrifuged to remove residual particles under 9,000 rpm for 10 min. Finally, 15 ml supernatant of the centrifuged filtrate was collected for measuring the total organic carbon (TOC) concentration. The adsorption efficiency (ε) was calculated by the following equation:

$$\begin{array}{l} \varepsilon =\frac{{\;T_{0}-\;T}_{1}\;}{T_{0}}\times 100\% \end{array}$$

where *T*_0_ represents the TOC concentration of freshly prepared SA solution of 40 mg/L; *T*_1_ means the TOC concentration of the supernatant obtained from the adsorption tests.

### Zeta Potential Measurements

Zeta potentials of the molybdenite and chalcopyrite particles were measured using a JS94H micro electrophoresis instrument (Shanghai Zhongchen Digital Technic Apparatus Co., Shanghai, China) under the room temperature of 25°C. KNO_3_ solution of 1 × 10^−3^ mol/L was used as a supporting electrolyte. For each measurement, 20 mg pure mineral (−2 μm) was dispersed in 40 ml of the electrolyte, and the suspension was magnetically stirred for 2 min after pH adjustment. Then, flotation reagents of desired dosages were added per the abovementioned reagent schemes. After standing for 5 min, the supernatant was collected for zeta potential measurement. The measurement under each reagent condition was conducted at least three times, and the average values were accepted as the final results.

### FTIR Spectroscopy Analyses

FTIR analyses in this study were performed using a 740FT-IR instrument (Nicolet Co., MA, USA) with the diffuse reflection approach (30 scans, resolution 2 cm^−1^) under room temperature. Spectra were collected in the absorption band range of 400 to 4,000 cm^−1^. Tested samples were prepared by adding 1.0 g pure mineral particles (−2 μm) into 40 ml deionized water with a desired reagent scheme at pH 5.4. After conditioning for 30 min, the suspension was filtered and the filter cake was washed with deionized water of pH 5.4 for three times. Then, the filter cake was dried in a vacuum oven at 30°C. After the moisture got completely evaporated, the dry solid of 1 mg was mixed with 100 mg spectroscopic grade potassium bromide (KBr), and the mixture was used for the analysis.

## Results And Discussions

### Micro-Flotation Results

#### Single-Mineral Flotation Experiment Results

**Figure 3:**
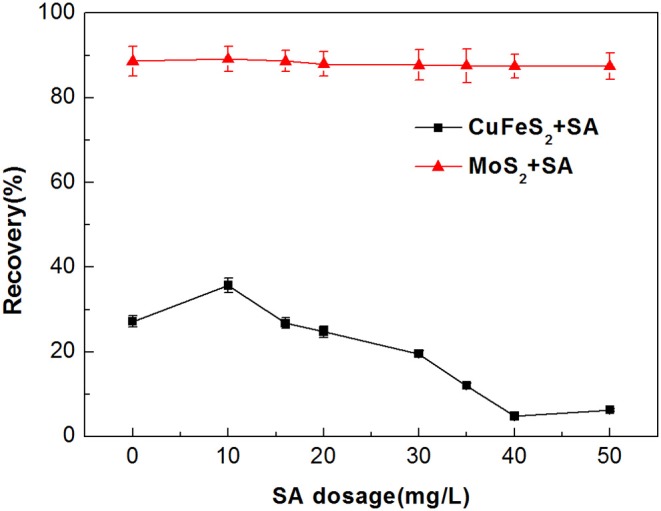
Flotation recovery of chalcopyrite and molybdenite as a function of sodium alginate (SA) dosage (2 mg/L kerosene and pH 5.4).

Figure 3 shows the effect of SA dosage on the flotation performance of molybdenite and chalcopyrite when using 2 mg/L kerosene as collector at pH around 5.4. It can be seen from the figure that chalcopyrite recovery was <5% when the dosage of SA exceeded 40 mg/L. Interestingly, high recoveries (nearly 90%) of molybdenite were maintained over the investigated SA dosage, indicating that SA imposed a minimal effect on the floatability of molybdenite. Therefore, the efficient separation of molybdenite from chalcopyrite is likely to be achieved by using SA and kerosene as depressant and collector, respectively.

**Figure 4:**
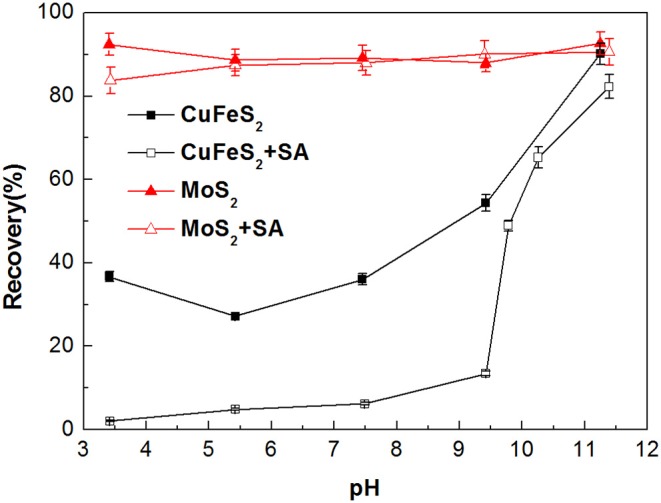
Flotation recovery of chalcopyrite and molybdenite as a function of pH with and without sodium alginate (SA) (40 mg/L SA and 2 mg/L kerosene).

The flotation recoveries of molybdenite and chalcopyrite as a function of pH in the absence and presence of SA are shown in Figure 4. The recovery of molybdenite changed slightly at the entire pH range with the addition of 40 mg/L SA, suggesting that SA barely influenced the molybdenite floatability. However, the recovery of chalcopyrite dropped significantly at pH 3–9 when 40 mg/L SA was added. Therefore, the optimum pH range for the separation of molybdenite from chalcopyrite was 3–9.

#### Mixed-Mineral Micro-Flotation Results

The aforementioned single-mineral flotation test results indicated that the efficient separation of molybdenite from chalcopyrite could be achieved by using SA as an inhibitor in the pH range of 3–9. When different minerals simultaneously appear in a solution, surface properties of the minerals can be greatly impacted by each other, resulting in changes in their affinities toward reagents (Jin et al., 2018; Tian et al., 2018). This can potentially reduce the selectivity of flotation reagents (Liu et al., 2016; Bicak et al., 2018; Feng et al., 2018). Therefore, flotation tests of mixed minerals (chalcopyrite plus molybdenite) were conducted to further investigate the selective inhibition performance of SA. The flotation results of a mixed-mineral system (the mass ratio of chalcopyrite to molybdenite equals 2:1) in the presence of 40 mg/L SA under pH 5.4 are presented in Table 1.

**Table 1:** The separation of chalcopyrite and molybdenite mixture [40 mg/L sodium alginate (SA) and pH 5.4].

**Test conditions**		**Products**	**Cu grade/%**	**Mo grade/%**	**Cu recovery/%**	**Mo recovery/%**
SA prior to kerosene	Kerosene dosage: 2 mg/L	Concentrate	21.64	28.98	23.42	35.97
		Tailing	22.02	16.05	76.58	64.03
		Feed	21.93	19.12	100	100
	Kerosene dosage: 19 mg/L	Concentrate	20.24	29.13	36.52	59.83
		Tailing	23.32	12.96	63.48	40.17
		Feed	22.10	19.40	100.00	100.00
kerosene prior to SA	Kerosene dosage: 19 mg/L	Concentrate	4.55	53.43	5.78	76.90
		Tailing	29.08	6.29	94.22	23.10
		Feed	22.17	19.57	100.00	100.00

When SA was added prior to kerosene of 2 mg/L and 19 mg/L, the concentrate Cu grades were 21.64 and 20.24%, respectively, indicating a weak selectivity of SA in the mixed-mineral system. Meanwhile, the recovery of Mo in tailing were 64.03% for 2 mg/L kerosene and 40.17% for 19 mg/L kerosene. Therefore, SA showed an inhibitory effect on molybdenite in the presence of chalcopyrite.

In order to eliminate the adverse effect of chalcopyrite on the flotation performance of SA, the effect of reagent scheme on the flotation results of chalcopyrite–molybdenite mixed systems was investigated. As shown in Table 1, a concentrate with Mo grade of 53.43% and recovery of 76.90% was acquired when kerosene was added before SA. This indicated that the addition of kerosene prior to SA could eliminate the adverse effect of chalcopyrite on the depressing effect of SA on molybdenite. Wie and Fuerstenau (1974) reported that adsorption of dextrin was reduced when molybdenite was coated with a layer of non-polar oil. This agrees with the findings from this study.

### Zeta Potential Measurement Results

**Figure 5:**
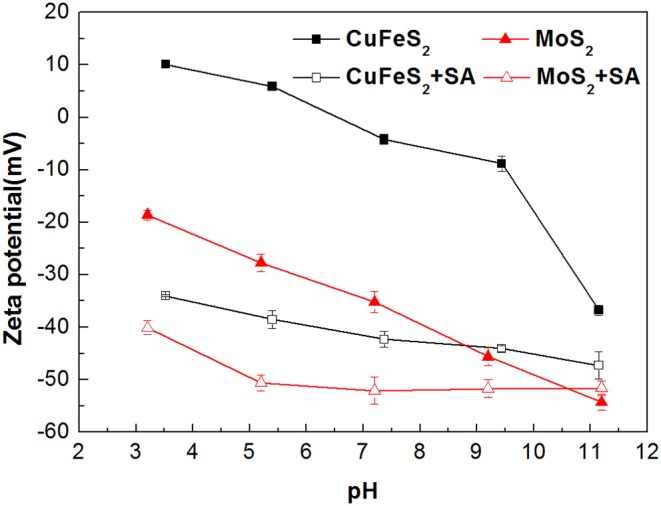
The zeta potentials of chalcopyrite and molybdenite before and after conditioning with sodium alginate (SA) at varied pH (40 mg/L SA and 2 mg/L kerosene).

Zeta potentials of chalcopyrite and molybdenite as a function of pH are presented in Figure 5. It can be observed that remarkable negative shifts occurred in the zeta potentials of chalcopyrite and molybdenite when SA was added alone. Moreover, the shift for chalcopyrite was larger than the molybdenite, suggesting that SA adsorbed more strongly on the chalcopyrite surface relative to the molybdenite at the tested pH range. This can be explained by the fact that more negative charges occur on the molybdenite surface relative to chalcopyrite (Reyes-Bozo et al., 2015; Castro et al., 2016; Han et al., 2019; Yang et al., 2019), resulting in a stronger electrostatic repulsion between molybdenite and SA (anionic agents). Moreover, metal cations dissolved from the chalcopyrite surface could also contribute to the higher adsorption of SA (Ikumapayi et al., 2012). It is noteworthy that when pH exceeded 9.5, the zeta potential difference of raw and SA-conditioned chalcopyrite decreased, which corresponded to the steep rise of chalcopyrite recovery (Figure 4).

### Fourier-Transform Infrared Spectra

**Figure 6:**
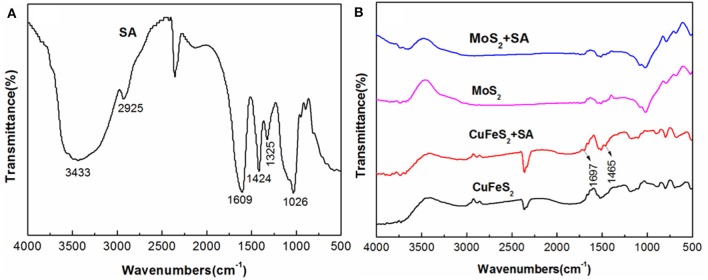
Fourier-transform infrared spectroscopy (FTIR) spectra of **(A)** sodium alginate (SA), **(B)** chalcopyrite and molybdenite (the SA treatment was performed by conditioning with 40 mg/L SA at pH 5.4).

Figure 6 presents the FTIR spectra of chalcopyrite and molybdenite before and after treatment with 40 mg/L SA at pH 5.4. The peaks at 1,609 and 1,424 cm^−1^ of the SA spectrum shown in Figure 6A were due to the stretching bands of -COO- (Rath et al., 2000; Chen et al., 2017, 2018), and the peak at 1,026 cm^−1^ originated from the stretching vibration of C-O-C. As shown in Figure 6B, after conditioning with SA, new peaks at 1,697 and 1,465 cm^−1^ occurred in the spectrum of chalcopyrite, which were shifted significantly compared to the corresponding peaks that present in the spectrum of SA (i.e., 1,609 and 1,424 cm^−1^). This proved that SA was chemically adsorbed on chalcopyrite surfaces and probably in the form of SA–Cu chelate, which has been reported in prior studies (Zhu et al., 2003; Liu et al., 2008). It is interesting to note that the spectrum of molybdenite treated by SA was barely changed relative to the raw specimen. Therefore, the adsorption of SA on molybdenite was negligible.

### Interaction Mechanisms of Chalcopyrite on the Sodium Alginate Performance in Molybdenite Flotation

#### The Effect of Sodium Alginate on the Flotation of Cu^2+^-Treated Molybdenite

**Figure 7:**
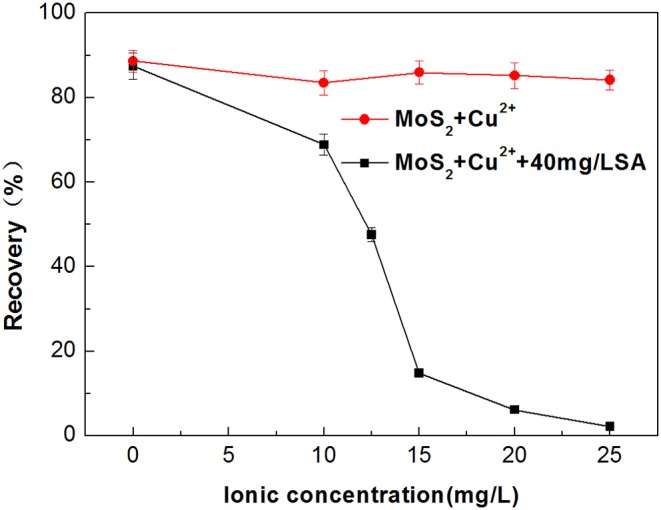
Flotation recovery of molybdenite with and without 40 mg/L sodium alginate (SA) as a function of Cu^2+^ initial concentration at pH 5.4.

**Figure 8:**
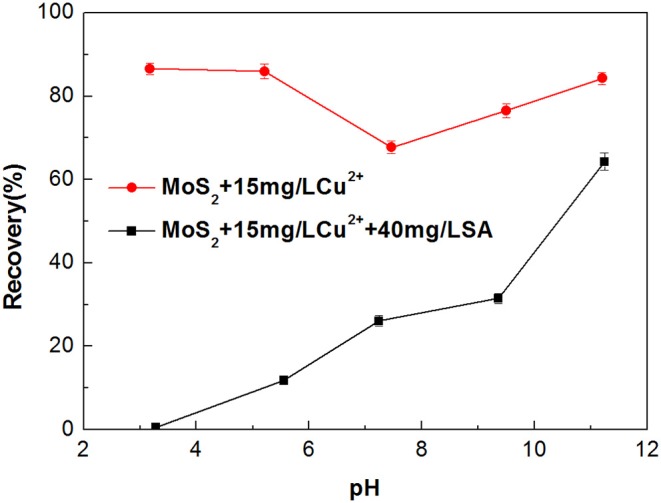
Flotation recovery of molybdenite with and without 40 mg/L sodium alginate (SA) as a function of pH in the presence of 15 mg/L Cu^2+^.

Metal ions dissolved from minerals may impose an adverse impact on the selectivity of flotation collector and depressant (Zhang et al., 2017; Zhang and Honaker, 2018). Dissolution of Cu^2+^ from the chalcopyrite surface is inevitable in the flotation separation of molybdenite from chalcopyrite (Zhao et al., 2018; Yang et al., 2019). Therefore, the effect of Cu^2+^ on the flotation performance of molybdenite was evaluated in the presence and absence of SA at pH around 5.4. As shown in Figure 7, when Cu^2+^ concentration varied from 0 to 25 mg/L, the recovery of molybdenite decreased significantly in the presence of 40 mg/L SA. Furthermore, the depressing effect of SA on molybdenite flotation was largely dependent on pH, and stronger inhibition occurred under acidic conditions (Figure 8).

#### Adsorption Characteristics of Sodium Alginate on Cu^2+^-Treated Molybdenite

**Figure 9:**
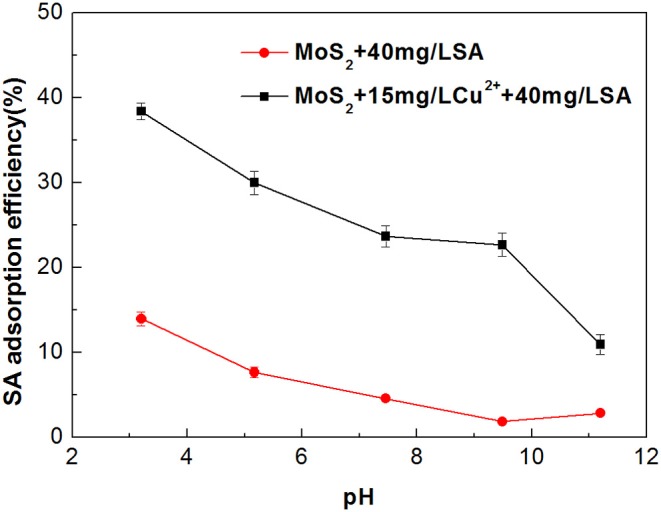
Adsorption efficiency of sodium alginate (SA) on molybdenite surfaces with and without 15 mg/L Cu^2+^ as a function of pH.

The impact of Cu^2+^ on the adsorption characteristics of SA on the molybdenite surfaces is shown in Figure 9. In the absence of Cu^2+^, much stronger adsorption occurred at relatively low pH, which can be explained by the formation of alginic acid that is insoluble under acidic conditions (Wang, 2007). When Cu^2+^ was added, the adsorption of SA on the molybdenite surface was increased significantly throughout the studied pH range. This is likely due to the increased amount of adsorption sites on the molybdenite surface provided by Cu species (Yang et al., 2019).

#### Zeta Potentials of Cu^2+^-Treated Molybdenite With and Without Sodium Alginate

**Figure 10:**
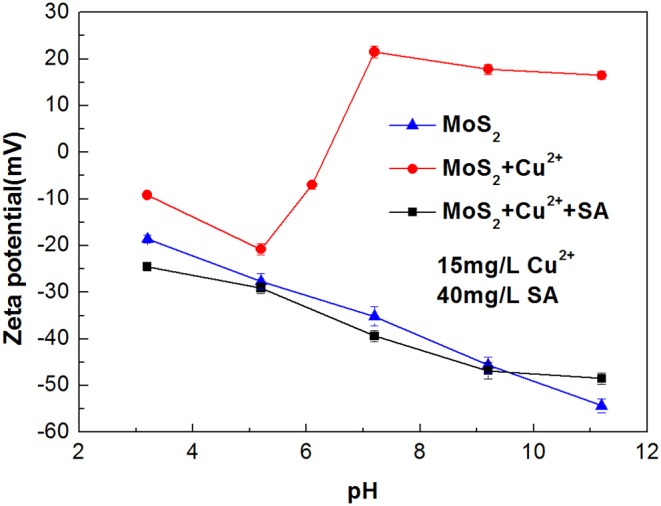
Zeta potentials of molybdenite at varied pH.

Zeta potentials of molybdenite in the presence and absence of Cu^2+^ with 40 mg/L SA were measured at different pH, and the results are presented in Figure 10. In the whole tested pH range, the raw molybdenite particles carried negative charges, and the zeta potential gradually decreased with the increase in pH, which agreed well with prior studies (Zhao et al., 2018). When the particles were dispersed in Cu^2+^ solution of 15 mg/L, the zeta potential of molybdenite increased and the trend curve was similar to that of Yang et al. (2019). According to their study, at pH lower than 5.4, the slightly positive shift of the molybdenite surface potential was due to a small amount of Cu^2+^ and Cu(OH)^+^ being adsorbed on the molybdenite surface which offset the partially negative charge. The sharp rise in the range of pH 5.4 to 9.5 was due to an increased amount of cupric species adsorbed and/or precipitated on the molybdenite surface. When 40 mg/L SA together with 15 mg/L Cu^2+^ appeared in the solution, the zeta potential of molybdenite was decreased, which likely resulted from the adsorption of negatively charged alginate ions (Wang and Yang, 2014). Meanwhile, as the pH increased, chelation between SA and cupric species in the solution was also promoted (Zhu et al., 2003), which partially led to the reduction of SA adsorption on molybdenite surfaces (Figure 7). At pH higher than 9.5, Cu(OH)_2(s)_ was dominant Cu species and the amount of ${\text{Cu}(\text{OH})}_{3}^{-}$ and ${\text{Cu}(\text{OH})}_{4}^{2-}$ also gradually increased. As a result, the depressing effect of SA on molybdenite was further weakened due to enhanced electrostatic repulsion.

#### Fourier-Transform Infrared Spectroscopy Spectra of Cu^2+^-Treated Molybdenite With and Without Sodium Alginate

**Figure 11:**
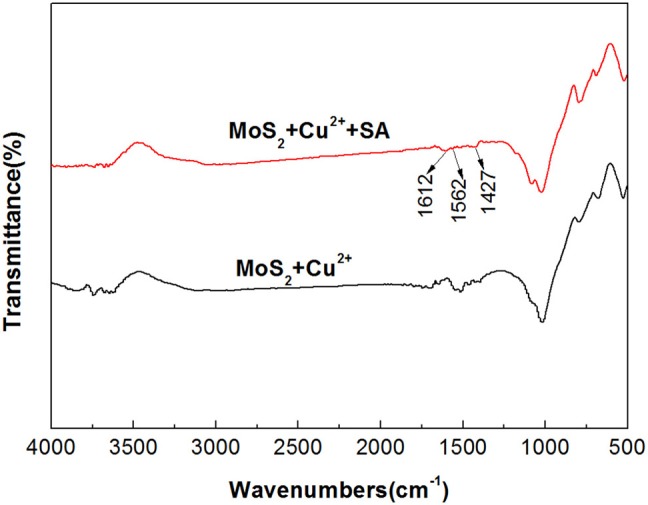
Fourier-transform infrared spectroscopy (FTIR) spectra of molybdenite with and without treatment of 40 mg/L sodium alginate (SA) in the presence of 15 mg/L Cu^2+^ at pH 5.4.

In order to further study the effect of Cu^2+^ on the depressing effect of SA on molybdenite, the IR spectra of molybdenite treated by Cu^2+^, Cu^2+^ + SA were measured and the results are presented in Figure 11. For molybdenite treated by Cu^2+^ and SA, new absorption bands at 1,612, 1,562, and 1,425 cm^−1^ were observed and the new bands corresponded to the stretching vibration of -COO- in SA, indicating the adsorption of SA on the molybdenite surface. The remarkable shift of the absorption band of -COO- from 1,609 to 1,562 cm^−1^ demonstrated that partial chemical adsorption of SA occurred on the molybdenite surface.

**Figure 12:**
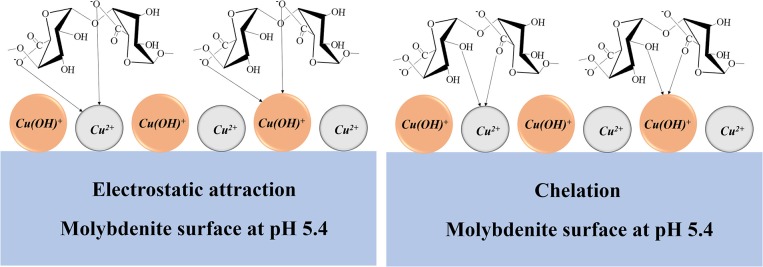
Schematic diagram of influence mechanism of chalcopyrite on the performance of sodium alginate (SA) in molybdenite flotation at pH 5.4.

Based on the results of the flotation tests and mechanism studies, a possible adsorption model was proposed to explain the depressing effect of chalcopyrite on the molybdenite flotation in the presence of SA at pH 5.4 (Figure 12). At pH 5.4, Cu ions occurring as Cu^2+^ and Cu(OH)^+^ adsorbed on the molybdenite surface. Meanwhile, SA existed in the form of alginic acid (a little) and alginate ions (dominantly), and the adsorption of SA on the molybdenite surface occurred by chelating with Cu species *via* the -COO- and -OH. The electrostatic attraction between the negative group -COO- and the Cu species also contributed to the adsorption of SA. The promotion of SA adsorption by Cu^2+^ resulted in the depression of molybdenite flotation.

## Conclusions

In this paper, interaction mechanisms of SA in the flotation separation of molybdenite from chalcopyrite were evaluated by micro-flotation tests, adsorption measurements, zeta potential measurements, and FTIR spectrum analyses. Single-mineral flotation tests showed that selective flotation separation of chalcopyrite and molybdenite could be achieved using SA as a depressant for the chalcopyrite. However, in the chalcopyrite–molybdenite mixed-mineral flotation system, the presence of chalcopyrite significantly increased the depressing effect of SA on the molybdenite floatability, reducing the selectivity of the separation. It is interesting to note that the addition of a certain dosage of kerosene prior to SA could restore the molybdenite floatability in the mixed-mineral flotation system. Given the findings of the adsorption tests, zeta potential measurements, and FTIR analyses, it was concluded that SA chemically adsorbed on the surface of chalcopyrite, thus causing a considerable decrease in its floatability. Moreover, strong adsorption of SA on the Cu^2+^-treated molybdenite surfaces also occurred in forms of electrostatic attraction and chelation. Therefore, the negative impact of SA on molybdenite flotation in the mixed-mineral system was due to the dissolution of Cu ions from the chalcopyrite surfaces which re-adsorbed onto the molybdenite surfaces and played as active sites for SA adsorption.

## Data Availability Statement

All datasets generated for this study are included in the article/supplementary material.

## Author Contributions

GZ and LO conceived the research, designed the tests, and analyzed the data. GZ, WZ, and YZ wrote and revised the manuscript.

## Conflict of Interest

The authors declare that the research was conducted in the absence of any commercial or financial relationships that could be construed as a potential conflict of interest.
